# Efficacy of polyphenols in adjuvant treating ulcerative colitis: A meta-analysis of randomized controlled trials

**DOI:** 10.1097/MD.0000000000041985

**Published:** 2025-05-23

**Authors:** Qiuxiang Wang, Liuying Li, Shuhan Zhuang, Mei Huang, Yongguo Xiang

**Affiliations:** a Department of Traditional Chinese Medicine, Guangyuan Central Hospital, Sichuan Province, China; b Department of Traditional Chinese Medicine, Zigong First People’s Hospital, Sichuan Province, China; c Zhejiang Chinese Medical University, Zhejiang Province, China; d The Second Hospital of Traditional Chinese Medicine of Yibin City, Sichuan Province, China.

**Keywords:** meta-analysis, polyphenols, randomized controlled trials, ulcerative colitis

## Abstract

**Background:**

The effectiveness and safety of polyphenols in treating ulcerative colitis remain controversial. This study aimed to evaluate the efficacy and safety of polyphenols in the treatment of ulcerative colitis.

**Methods:**

This study followed the preferred reporting items for systematic reviews and meta-analyses 2020 guidelines. A systematic search was conducted across PubMed, Embase, Web of Science, and Cochrane databases to identify relevant articles. The random-effects model was employed to calculate the odds ratio (OR) and corresponding 95% confidence intervals (CIs).

**Results:**

A total of 13 trials involving 742 participants were included in the meta-analysis. The clinical remission rates were higher in the polyphenol group compared to the control group, as demonstrated by both the intention-to-treat (ITT) (OR: 4.71; 95% CI: 2.02–10.99; *P* = .000) and per-protocol (PP) analysis (OR: 7.14; 95% CI: 3.11–16.39; *P* = .000). Similarly, the clinical response rate was higher in the polyphenol group compared to the control group, according to the ITT (OR: 5.40; 95% CI: 2.60–11.24; *P* = .000) and PP analysis (OR: 9.14; 95% CI: 4.25–19.64; *P* = .000). Moreover, the total endoscopic remission rate was superior in the polyphenol group, as indicated by the ITT (OR: 3.16; 95% CI: 1.20–8.37; *P* = .020) and PP analysis (OR: 4.92; 95% CI: 2.03–11.93; *P* = .000). No significant differences were observed regarding side effects between the 2 groups, according to the ITT (OR: 0.99; 95% CI: 0.56–1.76; *P* = .973) and PP analysis (OR: 0.99; 95% CI: 0.54–1.80; *P* = .971).

**Conclusion:**

Polyphenols demonstrated effectiveness in inducing clinical remission, clinical response, and endoscopic remission in patients with ulcerative colitis. Furthermore, there was no evidence of a higher incidence of adverse effects associated with their use.

## 1. Introduction

Ulcerative colitis (UC) is a chronic nonspecific inflammatory disease of the large intestine, characterized by continuous and diffuse inflammatory changes in colorectal mucosa.^[1]^ The condition primarily affects mucosa and submucosa, which belong to inflammatory bowel disease (IBD) in Western medicine.

UC incidence varies across countries, regions, and ethnic groups, demonstrating significant regional and ethnic differences. In Europe, the prevalence of UC in Norway is reported to be as high as 50.5/100,000,^[2]^ while in North America, the prevalence even exceeds 400/100,000.^[3]^ Epidemiological data from China indicate that UC prevalence was about 11.6 per 100,000 from 1990 to 2003.^[4]^ By 2023, the global prevalence of UC was estimated to be 5 million cases, with a sharp increase in global incidence.^[5]^

Individuals with UC face a shorter life expectancy and an increased risk of colectomy and colorectal cancer. In North America, Western Europe, and Oceania, the hospitalization rates for UC have remained stable, while in newly industrialized countries across Asia, Eastern Europe, and Latin America, the hospitalization rates are increasing rapidly, placing a growing burden on the global health care system.^[6]^ From 1990 to 2019, the overall disease burden of UC in China has increased significantly. Over these 30 years, both the incidence rate and disability life have been on the rise every year, regardless of gender and age.^[7]^ The direct and indirect expenses for patients with UC in China are about 65,000 yuan/year. However, the average reimbursement rate for most outpatient and inpatient expenses is <50%.^[8]^ Beyond the economic burden, UC is strongly associated with anxiety and depression, significantly affecting the patients’ quality of life and aggravating the social burden.^[9]^

Modern medicine primarily relies on immunosuppressants, glucocorticoids, biological agents, salicylic acid preparations, and other symptomatic treatments to relieve symptoms of UC. While these therapies can temporarily control the disease during use, symptoms often relapse upon discontinuation, leading to recurrent attacks of UC, reduced effectiveness after repeated treatments, and challenges in achieving complete clinical care. The curative effect of Western medicine and drugs aiming at single pathways and single targets in the treatment of UC is suboptimal, making the disease a global challenge in terms of treatment and drug development. There is an urgent need for new treatment ideas and methods to treat UC. New methods, such as polyphenols, are being tested to improve the therapeutic effect of UC.

Polyphenols are organic compounds composed of phenolic units that display a wide range of biological functions. Polyphenols such as curcumin, resveratrol, granatum, aloe vera gel, ginger powder, silymarin, and wheatgrass juice with potent anti-inflammatory, anticancer, and immunomodulatory properties can modulate key signaling molecules of enormous pharmacological interest.^[10]^ As immunomodulators, polyphenols have emerged as novel drug tools for treating various autoimmune diseases, including UC.^[11]^ However, the effectiveness and safety of polyphenols in treating UC remain controversial.^[12,13]^ To date, no known meta-analysis has been conducted on the treatment of UC by polyphenol compounds. Therefore, we conducted a meta-analysis of randomized controlled trials (RCTs) to evaluate the efficacy and safety of polyphenols in the treatment of UC.

## 2. Materials and methods

### 2.1. Inclusion and exclusion criteria

Two independent reviewers (QW and LL) reviewed the results of initial searches. RCTs published in English were included in our meta-analysis if they met the patients, intervention, comparator, outcome, and study design (PICOS) criteria. Patients: (1) participants aged 18 years or older; (2) with mild-to-moderately active or quiescent UC. The age range of the patients was 18 to 70 years old. The evaluation of UC activity was based on the Ulcerative Colitis Disease Activity Index (UCDAI score), Mayo score, Simple Clinical Colitis Activity Index (SCCAI), or Lichtiger Colitis Activity Index (LCAI score). The specific selection criteria are outlined in Table 1. Intervention: polyphenols were used alone or in combination with mesalamine in the experimental group. Comparator: the control group received a placebo alone or in combination with mesalamine. Outcomes: (1) primary outcomes: clinical remission; (2) secondary outcomes: clinical response, endoscopic remission, and side effects.

**Table 1:** The characteristics of each included study.

Author/reference	Assess the activity of UC	Polyphenol group	Control group	The primary outcome	Secondary outcome measures	Laboratory index
Singla V et al ^[14]^	UCDAI score	NCB-02 (curcumin) enema, qd, 8 w	Placebo enema, qd, 8w	Clinical remission	Clinical response Endoscopic Remission Side effects	NA
Banerjee R et al^[15]^	Mayo score	BEC (curcumin) 50 mg, po, bid, mesalamine 4.8 g, po, qd mesalamine 1 g, rectal, qd	Placebo 50 mg, po, bid, mesalamine 4.8 g, po, qd mesalamine 1 g, rectal, qd	Clinical remission	Endoscopic Remission Clinical Response Side effects	NA
Sadeghi N et al^[16]^	Simple Clinical Colitis Activity Index (SCCAI)	Curcumin, 1500 mg, po, qd, 8 w	Placebo, 1500 mg, po, qd, 8 w	IBDQ-9 score SCCAI score	Quality of life Side effects	TNF-α;hs-CRP;ESR complete blood count
*Kedia S* et al^[13]^	UCDAI score	Curcumin, 150 mg, po, tid, 8 w Mesalamine 800 mg, po, tid, 8 w	Placebo, 150 mg, po, tid, 8 w Mesalamine 800 mg, po, tid, 8 w	Clinical remission	Clinical response mucosal healing and treatment failure Side effects	ESR;Hemoglobin; Total leukocyte count;Urea;Alanine aminotransferase; Total protein;Albumin
Hanai H et al^[17]^	Clinical Activity Index	Curcumin, 1 g, po, bid, 6 month SZ (1.0–3.0 g/day; median, 2.0 g/day) or mesalamine (1.5–3.0 g/day; median, 2.25 g/day)	Placebo, 1 g, po, bid, 6 month SZ (1.0–3.0 g/day; median, 2.0 g/day) or mesalamine (1.5–3.0 g/day; median, 2.25 g/day)	Clinical remission	Recurrence Rates Side effects	NA
Lang A et al^[18]^	Simple Clinical Colitis Activity Index (SCCAI)	Curcumin, 1.5 g, po, bid, 1 month	Placebo, 1.5 g, po, bid, 1 month	Clinical remission	Clinical response Endoscopic Remission Side effects	NA
Samsamikor M et al^[19]^	Simple Clinical Colitis Activity Index (SCCAI)	Resveratrol, 500 mg, po, qd, 6 w	Placebo, 500 mg, po, qd, 6 w	The SCCAI score	The IBDQ-9 score Side effects	SOD;TCA;MDA
Samsamikor M et al^[20]^	Simple Clinical Colitis Activity Index (SCCAI)	Resveratrol, 500 mg, po, qd, 6 w	Placebo, 500 mg, po, qd, 6 w	The SCCAI score	The IBDQ-9 score Side effects	TNF-a;COX-2;NF-kB
Kamali M et al^[21]^	Lichtiger Colitis Activity Index	Granatum, 6 g, po, qd, 4 w	Placebo, 6 g, po, qd, 4 w	LCAI score	Change in each of the symptoms included in the LCAI side effects	NA
Langmead L et al^[22]^	Simple Clinical Colitis Activity Index (SCCAI)	Aloe vera gel, 100 mL, po, bid, 4 w	Placebo, 100 mL, po, bid, 4 w	Clinical remission Sigmoidoscopic remission histological remission	Simple Clinical Colitis Activity Index Baron score, histology score	Hemoglobin, platelet count, erythrocyte sedimentation rate, C-reactive protein and albumin
Nikkhah-Bodaghi M et al^[23]^	Simple Clinical Colitis Activity Index (SCCAI)	Ginger powder, 1 g, po, bid, 12 w	Placebo, 1 g, po, bid, 12 w	The index of disease activity SCCAI score	The quality of life questionnaire score	MDA, TAC
Rastegarpanah M et al^[24]^	The disease activity index (DAI)	Silymarin, 140 mg, po, qd, 6 month	Placebo, 140 mg, po, qd, 6 month	Clinical remission	Side effects	NA
Ben-Arye E et al^[25]^	Symptom diary, sigmoidoscopy, subjective improvement scale	100 cc of wheat grass juice, po, qd, 1 month	100 cc of placebo juice, po, qd, 1 month	Clinical remission	Side effects	NR

DAI = The disease activity index, LCAI score = Lichtiger Colitis Activity Index, NA = not applicable, NR = not reported, SCCAI score = Simple Clinical Colitis Activity Index.

Studies were excluded based on the following criteria: (1) duplicate articles; (2) articles published as observational studies, narrative reviews, meta-analyses, retrospective studies, case reports, animal experimental research, or conference presentations; (3) studies with data that cannot be extracted or lacked adequate data; (4) NOS scores of <5 points; (5) studies involving patients with other IBDs, such as Crohn disease; (6) studies in which no outcome measures of interest were reported.

### 2.2. Search strategy

This systematic review and meta-analysis were conducted in accordance with the Preferred Reporting Items for Systematic Reviews and Meta-Analyses 2020 guidelines^[26]^ and were registered in PROSPERO under the registration number CRD42023469960. A systematic search strategy was implemented across the following 4 databases with a time restriction from inception to October 2023 to identify the eligible studies: PubMed, Embase, The Cochrane Library, and Web of Science. The 2 sets of search terms used included “Ulcerative Colitis” and “Polyphenols.” For the articles updated multiple times, the latest or most complete version was included. The detailed search strategies are illustrated in Appendix A, Supplemental Digital Content, https://links.lww.com/MD/O984. The search keywords included: [Ulcerative Colitis] and [Polyphenols] or [Curcumin] or [Resveratrol] or [granatum] or [aloe vera gel] or [ginger powder] or [Silymarin] or [Wheat grass juice]. The flowchart depicting the study selection procedure is presented in Figure S1, Supplemental Digital Content, https://links.lww.com/MD/O985.

### 2.3. Data extraction

Two reviewers (MH and QW) independently extracted data from the included studies using a predesigned data extraction tool. Any discrepancies were resolved through negotiation and discussion. Further controversies were arbitrated by a third reviewer (YX). The following information was extracted from each included study: (1) study baseline (the first author’s name, published year, country, study design, polyphenol type, sample size, age, gender, UC severity and activity, polyphenols group regimen, and control group regimen); (2) primary outcomes: clinical remission; (3) secondary outcomes: clinical response, endoscopic remission, and side effects. The data characteristics are presented in Table 1.

### 2.4. Quality assessment

Two authors (LL and MH) independently assessed the risk of bias for each included article following the guidelines outlined in the Cochrane Handbook for Systematic Reviews of Interventions.^[27]^ The methodological quality was evaluated based on the following 7 domains: random sequence generation, allocation concealment, blinding of participants and personnel, blinding of outcome assessments, incomplete outcome data, selective reporting, and other biases. The risks were categorized as low, high, or unclear and displayed in graphical form. The quality assessment of each paper is detailed in Figure S2, Supplemental Digital Content, https://links.lww.com/MD/O986.

### 2.5. Statistical analysis

The Stata software (version 16.0, Stata Corp LP, College Station) was employed for meta-analysis and statistical analysis. For dichotomous data, the results are presented as odd ratios (ORs) with 95% confidence intervals (CIs). For continuous data, the results are reported as mean differences with 95% CIs. A *P* < .05 was considered statistically significant. Heterogeneity among studies was evaluated via chi-square tests and the inconsistency statistic.^[28]^ For studies reporting outcomes in medians and interquartile ranges, the method described by Wan et al was used to estimate the mean and standard deviation.^[29,30]^ The random-effects model was used for all statistical analyses. Both intention-to-treat (ITT) and per-protocol (PP) analyses were conducted. Sensitivity analysis was performed using the leave-one-out method to assess the effect of individual studies on the pooled data. Publication bias was assessed using Egger test and funnel plots for the adverse effects, as there were 10 articles that reported this outcome.

## 3. Results

### 3.1. Literature search

From the 4 electronic databases, 549 research articles closely related to the topic were initially identified. After preliminary screening and review, 362 studies were excluded due to being duplicate records, and 120 studies were excluded based on their title or abstract. Moreover, after carefully reading, reviewing, and confirming the full-text content, 13^[13–25]^ studies were ultimately included in this meta-analysis. The flowchart depicting the study selection process is illustrated in Figure S1, Supplemental Digital Content, https://links.lww.com/MD/O985.

### 3.2. Characteristics of the studies included

The detailed characteristics of studies included in the meta-analysis are summarized in Table 1. In total, 13 articles involving 742 patients were included. Of these, 378 patients were from the polyphenol compounds group, and 364 were from the control group. All studies were RCTs. The standard deviation and the average age of the patients ranged from 32.7 ± 8.9 to 45.2 ± 15.9 years. The study duration varied from 4 to 24 weeks. Curcumin was the most widely studied polyphenol included in the literature,^[13–18]^ with dosages ranging from 0.1 to 3 g/day. The control group often received a placebo alone or in combination with mesalamine. Except for 2 studies,^[17,24]^ all other subjects were patients with active UC, most of whom had moderate to severe active UC. These studies were published between 2002 and 2021 and were conducted in the following regions: Iran (n = 6), India (n = 3), UK (n = 1), Japan (n = 1), Israel (n = 1), and 1 study conducted across Israel, China, and Cyprus. The detailed characteristics of the included studies are summarized in Table 1.

### 3.3. Risk of bias in included studies

The Cochrane risk of bias assessment tool was employed to evaluate the quality of the included studies.^[27]^ Regarding random sequence generation, 10 trials used appropriate generation methods with a low risk of bias. The random number sequences were generated using either computer software or a random number table. However, 3 trials^[19,20,24]^ did not clearly describe the randomization procedure. Ten of the studies described double-blinding methods. Seven studies described methods for allocation concealment as sealed envelopes or drug containers with identical shapes and serial numbers. However, the remaining studies did not describe their methods for allocation concealment. Thirteen studies reported complete outcome data. While a few trials experienced patient dropouts, the dropout numbers were balanced between the experimental and control groups, with no significant impact on the results. Besides, no factors affecting the test results were identified, and all studies were identified as “low risk” for the reporting bias. All articles were assessed as low risk for other biases, as there were no baseline differences between the experimental and the control group. The summary is presented in Figure S2, Supplemental Digital Content, https://links.lww.com/MD/O986.

### 3.4. Primary outcomes

#### 3.4.1. Clinical remission

**Figure 1.**
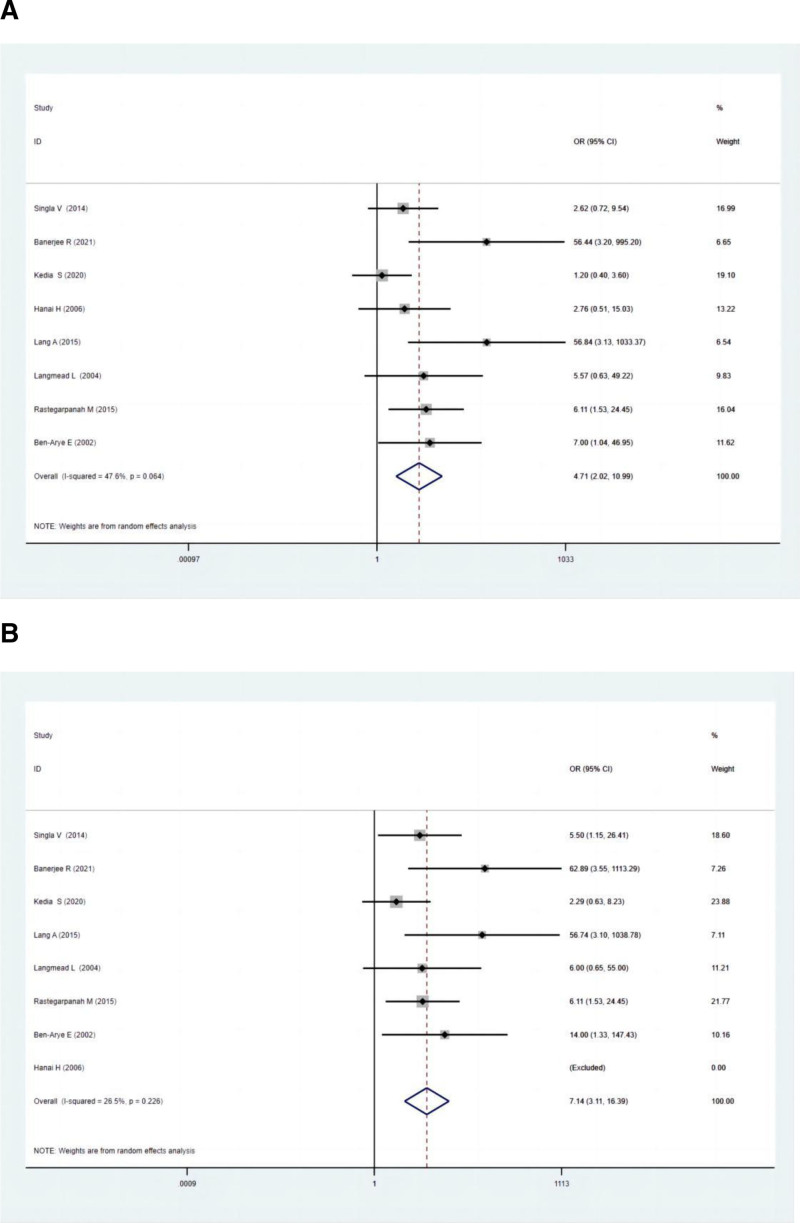
(A) Forest plots for clinical remission between experimental groups and control groups according to ITT analysis. (B) Forest plots for clinical remission between experimental groups and control groups according to PP analysis. ITT = intention-to-treat, PP = per-protocol.

Eight studies included in the meta-analysis compared the clinical remission rates between polyphenol and the control group. The control group was the placebo group, or the placebo combined with mesalazine. There was a significant difference in clinical remission between the 2 groups, as demonstrated by the ITT (OR: 4.71; 95% CI: 2.02–10.99; *P* = .000) and PP analysis (OR: 7.14; 95% CI: 3.11–16.39; *P* = .000) (Fig. 1A and B). The clinical remission with the polyphenol group demonstrated a significant, superior efficacy relative to those without polyphenol supplementation.

### 3.5. Secondary outcomes

#### 3.5.1. Clinical response rate

**Figure 2.**
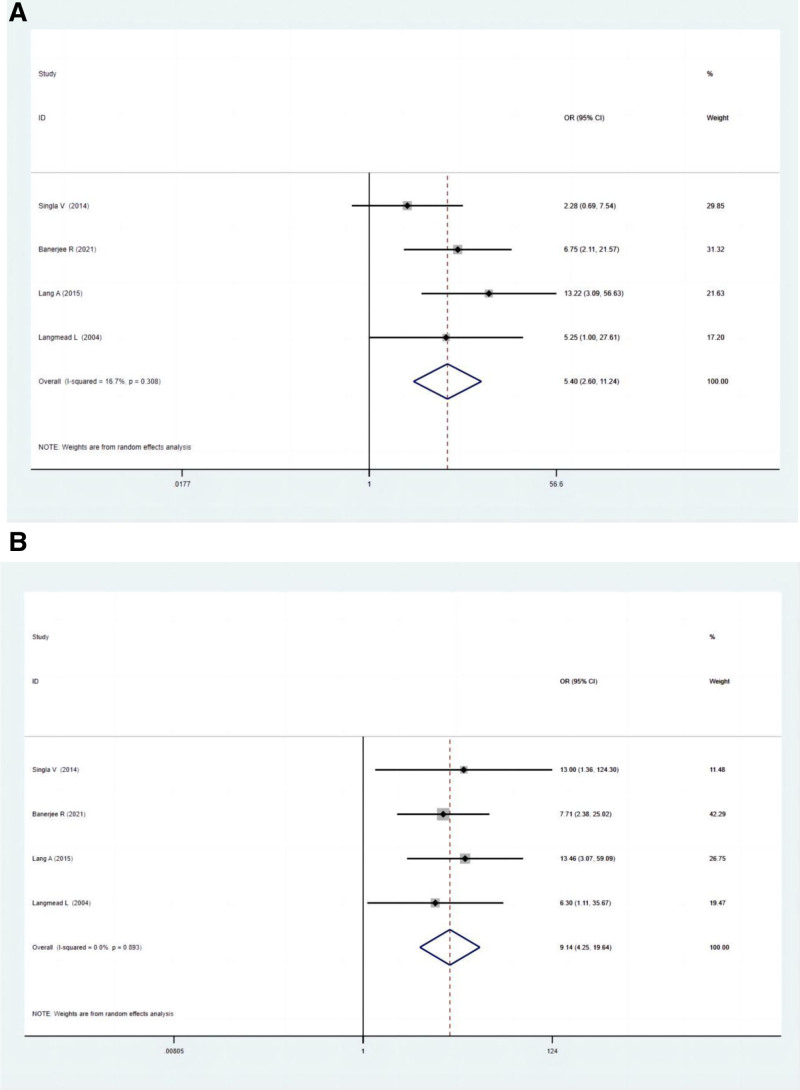
(A) Forest plots for clinical response rate between experimental groups and control groups according to ITT analysis. (B) Forest plots for clinical response rate between experimental groups and control groups according to PP analysis. ITT = intention-to-treat, PP = per-protocol.

Four studies compared the clinical response rate between polyphenol and the control group. There was a significant difference in clinical response rate between the 2 groups according to ITT (OR: 5.40; 95% CI: 2.60–11.24; *P* = .000) and PP analysis (OR: 9.14; 95% CI: 4.25–19.64; *P* = .000) (Fig. 2A and B). The clinical response rate with the polyphenol group indicated a significantly higher efficacy relative to those without polyphenol supplementation.

#### 3.5.2. Endoscopic remission

**Figure 3.**
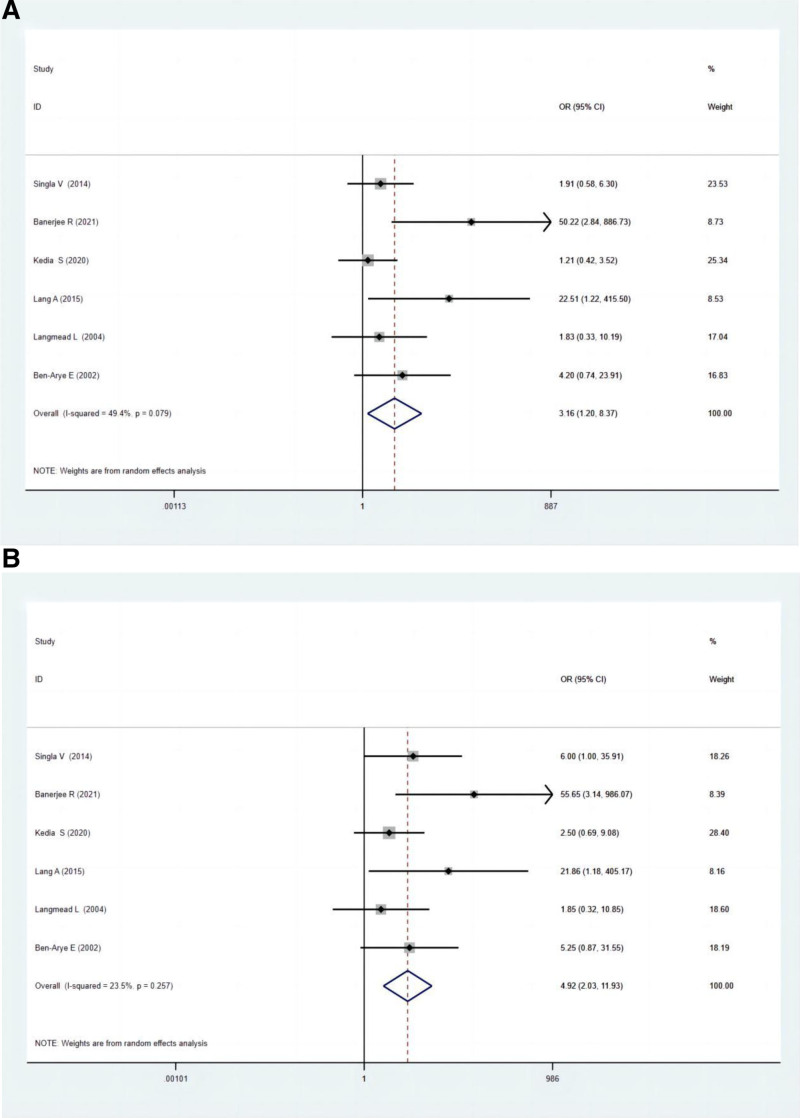
(A) Forest plots for endoscopic remission between experimental groups and control groups according to ITT analysis. (B) Forest plots for endoscopic remission between experimental groups and control groups according to PP analysis. ITT = intention-to-treat, PP = per-protocol.

Six studies compared the endoscopic remission between polyphenol and the control group. The total endoscopic remission rate in the polyphenol group was higher compared to the control group, as indicated by the ITT (OR: 3.16; 95% CI: 1.20–8.37; *P* = .020) and PP analysis (OR: 4.92; 95% CI: 2.03–11.93; *P* = .000) (Fig. 3A and B).

#### 3.5.3. Adverse effects

**Figure 4.**
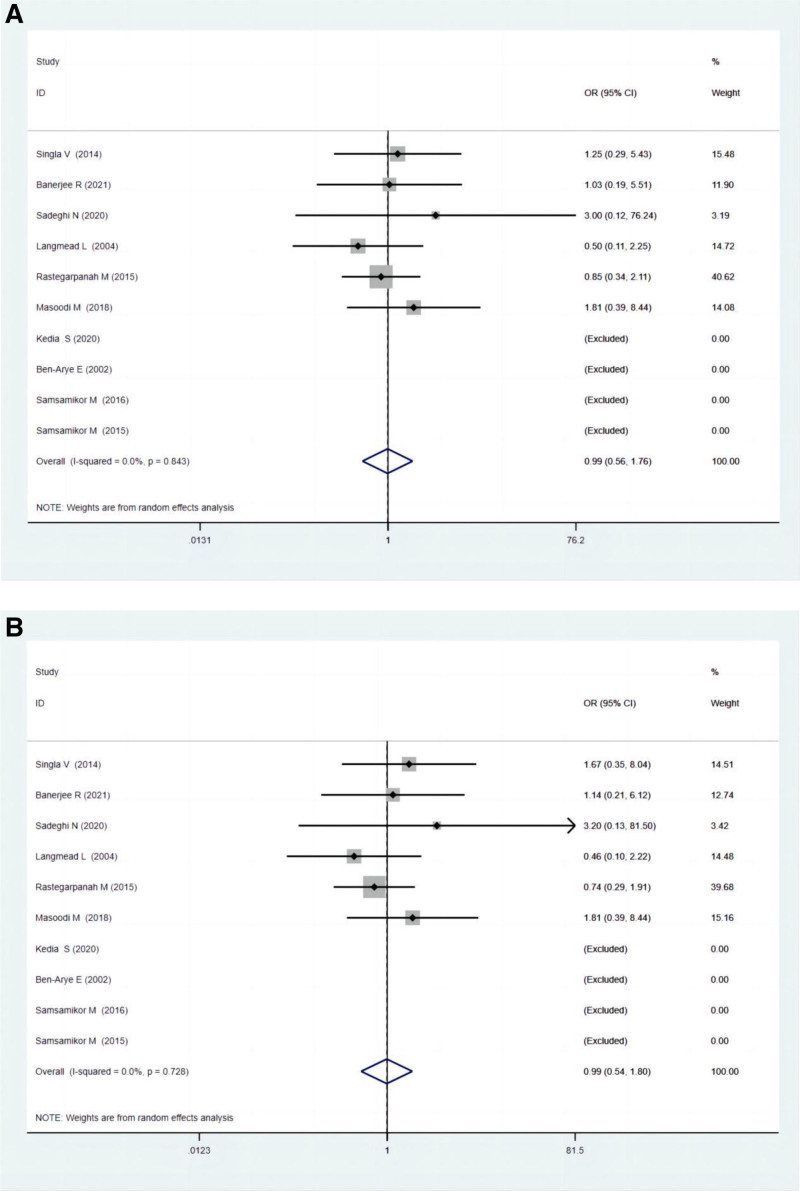
(A) Forest plots for adverse effects between experimental groups and control groups according to ITT analysis. (B) Forest plots for adverse effects between experimental groups and control groups according to PP analysis. ITT = intention-to-treat, PP = per-protocol.

Ten studies compared the endoscopic remission between polyphenol and the control group. There were no differences regarding adverse effects between the 2 groups, as demonstrated by the ITT (OR: 0.99; 95% CI: 0.56–1.76; *P* = .973) and PP analysis (OR: 0.99; 95% CI: 0.54–1.80; *P* = .971) (Fig. 4A and B).

### 3.6. Publication bias

Egger test for the side effects indicated no significant publication bias (*P* = 1.000). However, other outcomes were not analyzed for publication bias due to insufficient data (Figure S3, Supplemental Digital Content, https://links.lww.com/MD/O987).

### 3.7. Sensitivity analysis

By sequentially removing the individual studies, none altered the pooled data on the adverse effects (Figure S4, Supplemental Digital Content, https://links.lww.com/MD/O988). This indicates that the results of the meta-analysis are relatively reliable and stable.

## 4. Discussion

Ulcerative colitis is a chronic, disabling IBD. Various forms of mesalazine remain the standard treatment for uncomplicated UC.^[31]^ However, 20% to 30% of patients fail to respond to these drugs during induction of remission.^[32]^ Glucocorticoids are highly effective for the acute treatment of UC; however, their use is limited to short-term therapy due to their significant adverse effects.^[31]^ For patients who fail to respond to the initial therapy, immunomodulators and biologics are available as alternative management options.^[1]^ However, these medications are associated with mild-to-severe adverse effects such as infection, osteoporosis, depression, mutagenesis, bone marrow suppression, and malignancy. Clearly, the current therapy for UC is restricted by the limited efficacy and adverse effects of the commonly used therapeutic options.

Our research results indicate that polyphenol compounds significantly improve clinical remission, clinical response, and endoscopic remission rates compared to placebo. For clarity, it is important to note that in 3 of the included studies,^[13,15,17]^ both the intervention group (receiving polyphenols) and the control group were concomitantly taking mesalazine orally as part of their treatment regimen. In the remaining studies, the intervention group received only polyphenols, while the control group was administered a placebo, with no additional concomitant medications reported. These findings demonstrate the potential of polyphenol compounds as a promising therapeutic option for UC, offering a natural and potentially effective alternative or adjunct to conventional treatments. The consistent positive outcomes observed in comparison to placebo further strengthen the evidence supporting the efficacy of polyphenols in managing this condition.

Curcumin, a hydrophobic polyphenol, has been widely utilized in traditional Chinese medicine for treating inflammatory conditions since ancient times.^[33]^ A large number of studies have demonstrated that curcumin exhibits a variety of biological activities, including anti-inflammatory, anti-oxidation, and antitumor effects, with its anti-inflammatory effect being particularly notable.^[34]^ Patients with UC experience chronic and recurrent inflammation in the colon, resulting in excessive production of proinflammatory factors that damage the intestinal barrier. A large number of animal experiments have demonstrated that curcumin can alleviate DSS-induced colitis by regulating TLR4/NF-κB^[35]^ and Treg/Th17 signaling pathways,^[36]^ inhibiting NLRP3 flammable body activation and IL-1β production,^[37]^ and modulating autophagy and intestinal immunity.^[38]^ Furthermore, the therapeutic effects of curcumin on UC have been consistently demonstrated in clinical trials.^[14,16]^

Resveratrol (3,5,40-trihydroxytrans-stilbene) is a polyphenolic flavonoid natural compound known for its various beneficial effects on health, particularly in combating oxidative stress and inflammation.^[19]^ In experimental models of UC, resveratrol demonstrated the ability to improve the disease activity index, decrease oxidative stress, and lower inflammatory biomarkers while enhancing the activity of tissue antioxidant enzymes.^[20]^ Furthermore, several studies have revealed that 500 mg/day of resveratrol supplementation can improve the disease activity and quality of life in patients with UC by reducing oxidative stress and inflammation.^[19,20]^

Granatum accounts for 43% of the whole fruit with over 48 identified compounds, including polyphenols, which have antiproliferation, anti-inflammatory, and anticancer effects.^[39]^ Strong evidence indicates that polyphenol-rich pomegranate supplementation exerts anti-inflammatory effects that may ameliorate the symptoms of chronic inflammatory diseases such as IBDs in rodent models.^[40]^ A clinical trial has demonstrated that granatum peel extract appears to be effective in the complementary management of UC.^[21]^

Aloe is an ancient medicinal plant from the Asphodelaceae family, known for its bioactive constituents, such as polyphenols.^[41]^ Animal model studies have demonstrated that aloe vera can improve UC by enhancing colonic mucus barrier function and reducing inflammation.^[42]^ Moreover, a clinical trial revealed that oral aloe vera for 4 weeks was more likely to produce a clinical response than a placebo, and it also reduced the activity of histological diseases.^[22]^

Ginger has been identified to possess a variety of bioactive compounds, with its pharmacological effects primarily attributed to polyphenols.^[43]^ Oxidative stress plays a crucial role in the initiation and reoccurrence of UC. Oral gingerol can prevent DSS-induced chronic UC via anti-inflammatory and anti-oxidative mechanisms and preservation of the Wnt/β-catenin signaling pathway.^[44]^ Moreover, a clinical trial indicated that the consumption of dried ginger powder can improve certain aspects of oxidative stress and disease activity in patients with UC.^[23]^

Silymarin is a plant-derived mixture of polyphenolic flavonoids. Studies have revealed that silymarin increased the total antioxidant capacity of colonic tissue in TNBS-induced colitic rats. Additionally, treatment with silymarin in these rodents significantly decreased colonic NF-κB activity, levels of IL-1β, TNF-α, TBARS, and MPO activity.^[45]^ Moreover, silymarin can help patients with UC maintain a remission state, and the drug has been reported to be well tolerated.^[24]^

Wheatgrass juice, extracted from the pulp of wheatgrass, has been used for managing various intestinal diseases for several years. Flavonoids and polyphenols in wheat straw exhibit anti-inflammatory and antioxidant properties, which can alleviate inflammatory responses and oxidative stress. Apigenin, the main constituent in wheatgrass, has been demonstrated to block lipopolysaccharide-induced lethality in vivo and proinflammatory cytokine expression by inactivating NF-κB through the suppression of p65 phosphorylation.^[46]^ Moreover, the patients treated with wheatgrass juice experienced significant reductions in disease activity index and the severity of rectal bleeding.^[25]^

The advantages of this meta-analysis are as follows. Firstly, this study is the first meta-analysis to evaluate the efficacy and safety of polyphenols in UC treatment. The findings have significant clinical implications, as they provide evidence-based guidance on the use of polyphenols in the management of UC, potentially offering a natural and complementary treatment option. Secondly, the study employed a thorough and systematic literature search across multiple databases, ensuring that all relevant studies on polyphenols and UC were included, minimizing the risk of selection bias. Thirdly, the use of strict inclusion and exclusion criteria for selecting studies ensured that only high-quality, relevant research was included in the meta-analysis, enhancing the validity of the findings. Fourthly, sensitivity analyses were performed to evaluate the stability of the results, ensuring that the findings were not overly influenced by any single study or outlier. The study assessed potential publication bias using methods such as Egger test and funnel plots, which adds to the credibility of the meta-analysis by addressing potential biases in the literature. Additionally, the use of both ITT and PP analyses provided a more comprehensive evaluation of the treatment effects, accounting for different scenarios and increasing the reliability of the conclusions. However, this review has certain limitations. First, due to insufficient data, we were unable to analyze clinical remission, clinical response, and endoscopic remission based on the different types of polyphenols. Second, the number of cases included in this meta-analysis is small.

This meta-analysis offers valuable implications to guide clinical practice. Adding polyphenols such as curcumin, resveratrol, granatum, aloe vera gel, ginger powder, silymarin, and wheatgrass juice to the routine treatment of patients with UC is associated with significant symptom improvement, including reduced clinical activity of UC. Polyphenols have proven to be effective in the complementary management of UC, particularly in maintaining remission. Future research should focus on determining the optimal dosage of polyphenols for UC treatment.

## 5. Conclusion

This systematic review and meta-analysis revealed that the addition of polyphenols, including curcumin, resveratrol, granatum, aloe vera gel, ginger powder, silymarin, and wheatgrass juice, was effective in inducing clinical remission, clinical response, and endoscopic remission. However, no evidence of an increased rate of adverse effects was identified. However, they provide valuable insights that could assist clinicians in optimizing therapeutic strategies for managing mild-to-moderately active or quiescent UC.

## Author contributions

**Conceptualization:** Qiuxiang Wang.

**Formal analysis:** Qiuxiang Wang, Shuhan Zhuang.

**Investigation:** Liuying Li.

**Methodology:** Shuhan Zhuang, Mei Huang.

**Software:** Liuying Li.

**Supervision:** Yongguo Xiang.

**Writing – original draft:** Qiuxiang Wang.

**Writing – review & editing:** Qiuxiang Wang.

## Supplementary Material


