# Supplementary material for: Efficacy of polyphenols in adjuvant treating ulcerative colitis: A meta-analysis of randomized controlled trials
Source: Medicine (Baltimore). 2025 May 23;104(21):e41985. doi: 10.1097/MD.0000000000041985 (PMC12114046; doi:10.1097/MD.0000000000041985)
Supplement: Supplementary file 1 [file medi-104-e41985-s001.pdf]

Appendix A Search strategy in PubMed database
Search items
#1 ((Polyphenol[MeSH Terms]) OR (Polyphenol)) OR (Provinols)<br>#2 (((((Curcumin[MeSH Terms]) OR (1,6-Heptadiene-3,5-dione, 1,7-bis(4-hydroxy-3-methoxyphenyl)-, (E,E)-)) OR (Turmeric Yellow)) OR (Curcumin Phytosome)) OR (Diferuloylmethane)) OR (Mervia)<br>#3 (((((((((resveratrol[MeSH Terms]) OR (3,4',5-Stilbenetriol)) OR (3,5,4'-Trihydroxystilbene)) OR (3,4',5-Trihydroxystilbene)) OR (trans Resveratrol)) OR (Resveratrol-3-sulfate)) OR (SRT 501)) OR (cis-Resveratrol)) OR (Resveratrol, (Z)-)) OR (trans-Resveratrol-3-O-sulfate)<br>#4 (((((pomegranate fruit rind[MeSH Terms]) OR (granati pericarpium)) OR (pomegranate husk)) OR (Punica granatum fruit rind)) OR (Punica granatum pericarp extract)) OR (granati cortex)<br>#5 aloe vera gel<br>#6 ginger powder<br>#7 ((((silymarin[MeSH Terms]) OR (Silimarin)) OR (Carsil)) OR (Karsil)) OR (Legalon)<br>#8 wheat grass juice<br>#9 #1or #2 or #3or #4 or #5 or #6 or #7 or #8<br>#10 (((Colitis Gravis[MeSH Terms]) OR (Idiopathic Proctocolitis)) OR (Inflammatory Bowel Disease, Ulcerative Colitis Type)) OR

(Ulcerative Colitis)

#11 #9 and #10
