# Supplementary material for: Efficacy of polyphenols in adjuvant treating ulcerative colitis: A meta-analysis of randomized controlled trials
Source: Medicine (Baltimore). 2025 May 23;104(21):e41985. doi: 10.1097/MD.0000000000041985 (PMC12114046; doi:10.1097/MD.0000000000041985)
Supplement: Supplementary file 2 [file medi-104-e41985-s002.pdf]

**Supplementary material 1** Flow diagram of search strategy and study selection.

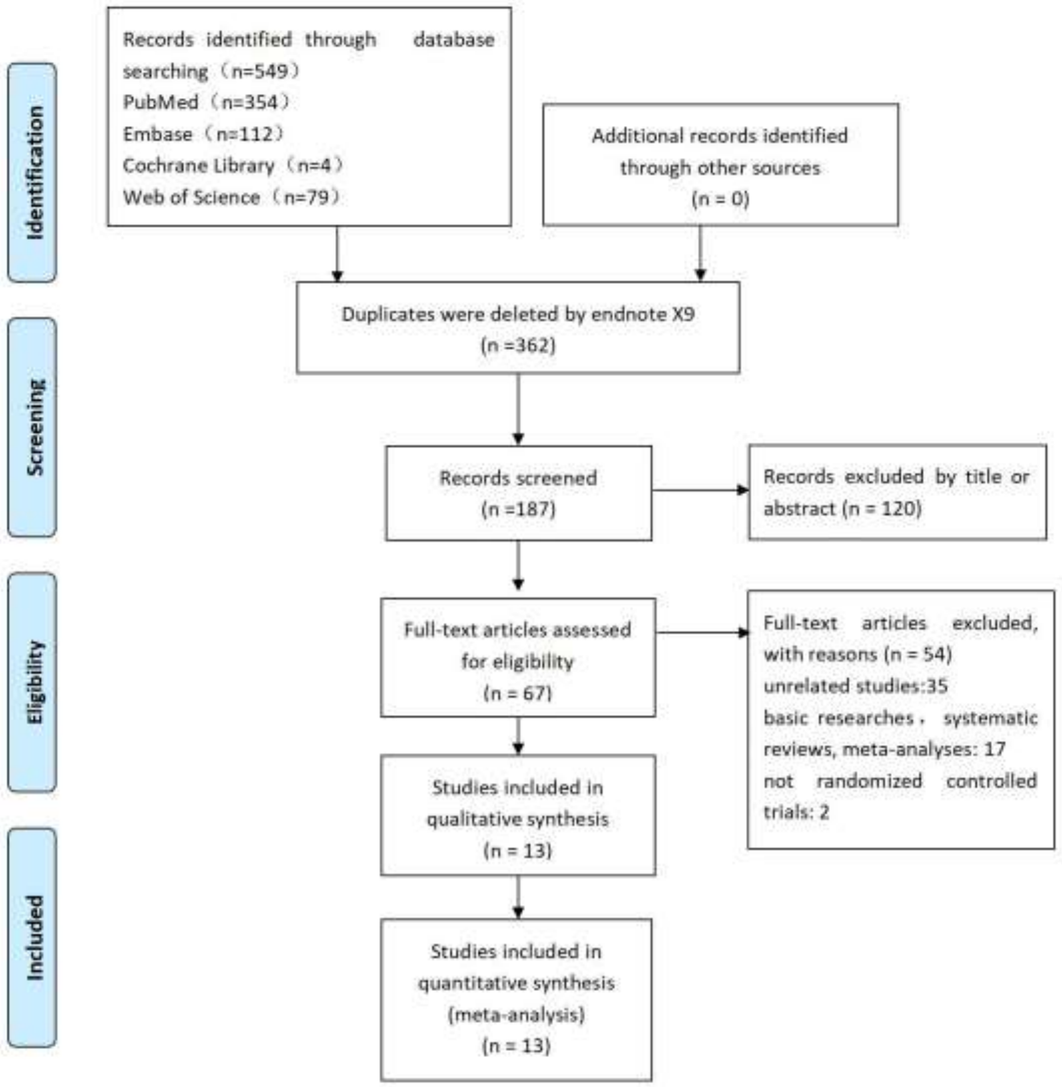


Figure 1 Flow diagram of search strategy and study selection.
