# Supplementary material for: Efficacy of polyphenols in adjuvant treating ulcerative colitis: A meta-analysis of randomized controlled trials
Source: Medicine (Baltimore). 2025 May 23;104(21):e41985. doi: 10.1097/MD.0000000000041985 (PMC12114046; doi:10.1097/MD.0000000000041985)
Supplement: Supplementary file 3 [file medi-104-e41985-s003.pdf]

Supplementary material 2 Risk of bias of the included randomized controlled trials

| Studies with intention-to-treat | Unique ID | Study ID | Experimental | Comparator | Outcome | Weight | Randomization process | Deviations from intended | Missing outcome data | Measurement of the outcom | Selection of the reportec | Overall |
|---|---|---|---|---|---|---|---|---|---|---|---|---|
| | A1 | Vikas Singla 2013 | NCB-02 (curcumin | placebo enema | clinical remissior | 1 | + | + | + | + | ? | + |
| | A2 | Banerjee R 2021 | BBC (curcumin) | placebo | clinical remissior | 1 | + | + | + | + | + | + |
| | A3 | Samsamikor M 2016 | Resveratrol | placebo | the SCCAI score | 1 | + | + | + | + | ? | + |
| | A4 | Samsamikor M 2015 | Resveratrol | placebo | the SCCAI score | 1 | + | + | + | + | + | + |
| | A5 | Sadeghi N 2020 | curcumin | placebo | IBDQ-9 score | 1 | + | + | + | + | ? | ? |
| | A6 | Kedia S 2017 | curcumin | placebo | clinical remissior | 1 | + | + | + | + | ? | + |
| | A7 | Hanai H 2006 | curcumin | placebo | clinical remissior | 1 | + | + | + | + | + | + |
| | A8 | Lang A 2015 | curcumin | placebo | clinical remissior | 1 | + | + | + | + | + | + |
| | A9 | Langmead L 2004 | aloe vera gel | placebo | clinical remissior | 1 | + | + | + | + | + | + |
| | A10 | Nikkhah-Bodaghi M | ginger powder | placebo | the index of dise: | 1 | + | + | + | + | ? | + |
| | A11 | Kamali M 2015 | granatum | placebo, | LCAI score | 1 | + | + | + | + | + | + |
| | A12 | Rastegarpanah M 2 | Silymarin | placebo | clinical remissior | 1 | + | + | + | + | ? | + |
| | A13 | Ben-Arye E 2002 | wheat grass juic | placebo juice | clinical remissior | 1 | + | + | + | + | ? | + |

+ Low risk

? Some concerns

? High risk
