# Supplementary figures and images for: Efficacy of polyphenols in adjuvant treating ulcerative colitis: A meta-analysis of randomized controlled trials
Source: Medicine (Baltimore). 2025 May 23;104(21):e41985. doi: 10.1097/MD.0000000000041985 (PMC12114046; doi:10.1097/MD.0000000000041985)

**Supplementary material 3** The funnel plot of adverse effects

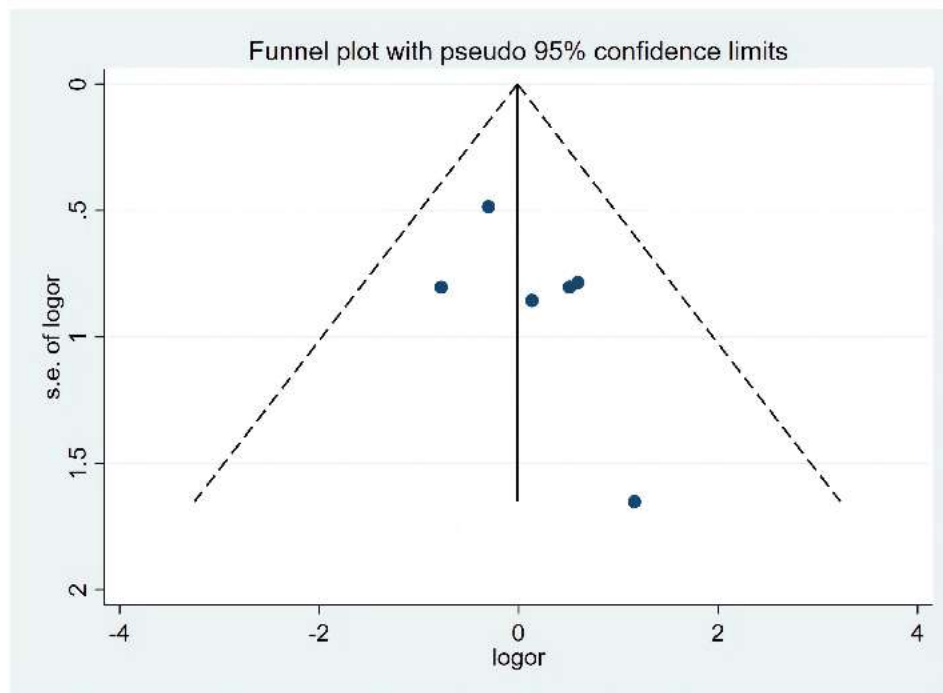

Supplement: Supplementary file 4 [file medi-104-e41985-s004.pdf]

## Supplementary material 4 Sensitivity analysis

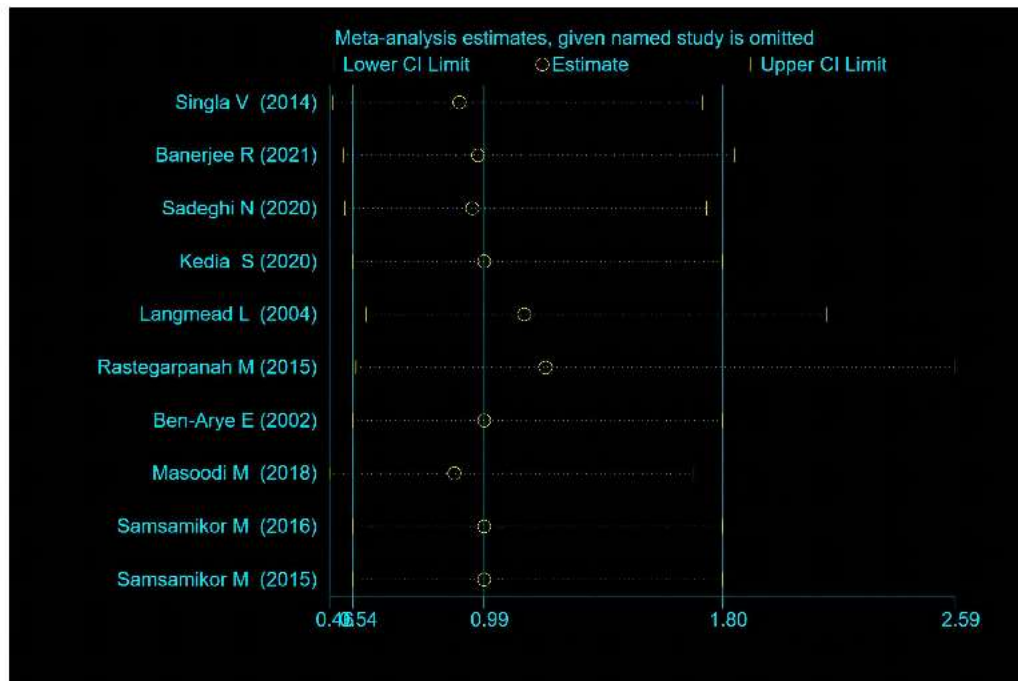

Supplement: Supplementary file 5 [file medi-104-e41985-s005.pdf]
